# Interorganizational Networks in Physical Activity Promotion: A Systematic Review

**DOI:** 10.3390/ijerph18147306

**Published:** 2021-07-08

**Authors:** Irina Timm, Simone Rapp, Christian Jeuter, Philip Bachert, Markus Reichert, Alexander Woll, Hagen Wäsche

**Affiliations:** 1 Mental mHealth Lab, Chair of Applied Psychology, Institute of Sports and Sports Science, Karlsruhe Institute of Technology (KIT), 76131 Karlsruhe, Germany; markus.reichert@rub.de; 2 Institute of Sports and Sports Science, Karlsruhe Institute of Technology (KIT), 76131 Karlsruhe, Germany; simone.rapp9@kit.edu (S.R.); christian.jeuter@gmx.de (C.J.); philip.bachert@kit.edu (P.B.); alexander.woll@kit.edu (A.W.); hagen.waesche@kit.edu (H.W.); 3 Central Institute of Mental Health (CIMH), Department of Psychiatry and Psychotherapy, Medical Faculty Mannheim, Heidelberg University, 68159 Mannheim, Germany; 4 Department of eHealth and Sports Analytics, Ruhr-University Bochum, 44801 Bochum, Germany

**Keywords:** social network analysis, physical activity, health promotion, public health, community networks, exercise

## Abstract

Public health challenges such as physical inactivity are multiplex and cannot be effectively addressed by single organizations or sectors. For this reason, public health policies have to involve various sectors and foster partnerships among organizations. Social network analysis (SNA) provides a methodological toolkit that enables the investigation of relationships between organizations to reveal information about the structure and cooperation within networks. This systematic review provides an overview of studies utilizing SNA to analyze the structure of networks that promote physical activity, including the structural set-up, types, and conditions of cooperation, the existence or absence of key actors, the characteristics of organizations working together, and potential barriers limiting collaboration. In total, eight eligible studies were identified. To evaluate the quality of these studies, a quality assessment tool for SNA was created. Relevant aspects from each study were systematically outlined using a data extraction template developed for network studies. The studies reported low to moderate density scores with many ties not being realized. Organizations tend to work side by side than as real partners, whereas organizations of the same type are more strongly connected. Most of the studies identified governmental health organizations as key players in their networks. Network maturity influences network outcomes. Shared goals and geographic proximity are potential facilitators for network development. For future research, more sophisticated methods and longitudinal studies are required to describe how networks, with the aim of promoting physical activity, develop and change to identify predicting factors for an effective network structure.

## 1. Introduction

Insufficient physical activity (PA) and sedentary behavior are key risk factors for non-communicable diseases (NCDs) such as diabetes, cancer, cardiovascular diseases, and are leading risk factors for global mortality [1,2,3,4]. Regular moderate PA can significantly improve health and counteract NCDs [5,6]. To this end, public health systems, policies, and programs promoting an active lifestyle are required [7]. This includes the integration of PA into daily routines, e.g., at work, home, school, and policies that help to reduce barriers to active transport (e.g., walking, bicycling), improve access to sports and recreational facilities, and induce a long-term change in health behavior [8,9,10,11]. Because of the strong link between PA and NCDs, the World Health Organization (WHO) member states agreed upon aiming to reduce insufficient PA by 10% by 2025 [12]. However, a study on worldwide trends regarding reduced PA showed that levels of insufficient PA are stable with even an increase in high-income countries [1]. To reverse the trend, public health systems are required that effectively address low levels of PA [13].

Public health systems are based on cooperation and partnership among various stakeholders (e.g., [14,15,16]). Hence, the development and implementation of effective interorganizational networks among stakeholders are critical in public health practice and PA promotion [17,18]. Stakeholder cooperation provides various benefits such as information exchange, knowledge sharing, the mobilization and leveraging of new resources, a generation of greater public awareness and support, and the creation of a critical mass for action [17,19].

To analyze interorganizational networks, social network analysis (SNA) can be applied. SNA originated in sociology, has a tradition in many different research disciplines (psychology, political science, business, mathematics), and is now increasingly being used in public health, sports, and sport management research [20,21,22,23,24,25,26,27].

SNA is defined as the analysis of relations (ties) that link elements or actors (nodes) within a network [28,29,30]. SNA is a combination of theory and methods that allows researchers to investigate and understand relations (e.g., information flow, collaboration, competition) among social actors (e.g., persons or organizations). In terms of promoting PA, relationships between organizations have often been studied in the past [31,32]. The shared resources between actors through their relations can be both tangible and intangible [31].

According to Luke and Harris, network measures in public health are structured to an individual, subgraph, and network level [26]. At the network level, which focuses on the entire system of nodes and links, characteristics such as density and centralization can be measured. Density describes the overall level of connectedness among organizations in the network and thus is a key descriptor of network structure [33,34]. Its value ranges from 0 to 1, where 1 means the network is completely connected. It is calculated as the number of connections compared to the total possible number of connections [32]. Centralization illustrates the extent to which the graph shows a hierarchical or centralized structure [26,35].

At the individual level, centrality identifies the position and characteristics of an actor within a network [26,36]. Centrality illustrates which organizations are most central or most involved in the network [37]. It is defined by the number of ties connected to a given actor; hence, most central organizations have the greatest number of links or ties to others [38]. There are various indicators of centrality; betweenness centrality describes the extent to which an actor lies on the shortest path between any other two nodes [26]. Since there is control of information flow, organizations with high betweenness centrality scores may act as gatekeepers in the network [39,40]. Closeness centrality indicates the average distance between one actor to all other actors. If actors play similar roles within a network by having the same patterns of connections to other actors, this is referred to as structural equivalence [26].

Besides numerical and descriptive analysis, statistical network analysis allows for investigating the formation and effects of networks [41,42,43]. To verify hypotheses involving network characteristics, techniques such as exponential random graph models (ERGM) can be used [44].

Therefore, SNA provides a powerful methodological toolkit to analyze positions (e.g., centrality) or roles (e.g., structural equivalence) of individual actors in networks; it can be used to evaluate the interorganizational structure of public health systems and assess the effectiveness of public health networks [45,46]; it allows researchers to illustrate and analyze the connections among organizations, to identify roles, subgroups, central and isolated actors, and to describe gaps and barriers within the network, thus supporting an understanding of collaborative relationships between organizations promoting PA [47]. Additionally, SNA allows researchers to measure the characteristics of partnerships, i.e., to reveal the content and strength of ties, to evaluate collaboration and effectiveness in organizations, and illustrate ways of communication [48].

This study focuses on the use of SNA in interorganizational networks targeting the promotion of PA. In particular, this review addresses a gap in extant literature: to date, to the best of our knowledge, this is the first systematic literature analysis summarizing the current state of research on interorganizational networks that promote PA. Therefore, specific templates for quality assessment (QA) and data extraction (DE) for network analyses were developed. To promote health through PA, it is critical to understand the structure and function of PA promoting networks. This review’s main purpose is to summarize the knowledge gained by studies that applied SNA to analyze the structure of PA promoting networks and discuss key findings. In particular, there are two aims. First, we aim to explore the structural characteristics and mechanisms of interorganizational networks promoting PA. Second, we focus on individual actors of these networks, such as key actors, characteristics of involved organizations, and potential barriers to cooperation. Furthermore, network maturity and its influence on network structure are scrutinized. Finally, the current and future utilization of SNA in this research field is discussed, and practical implications for effective network development and management to promote PA are provided.

## 2. Materials and Methods

This systematic review is based on a comprehensive search of relevant studies following defined eligibility criteria. Identified studies were evaluated and categorized. The review was conducted following the PRISMA checklist [49].

### 2.1. Eligibility Criteria

To be included in the review, studies had to meet the following eligibility criteria: (1) used network analysis methods. Due to the novelty of this research area, the review was designed to be as inclusive as possible—therefore, studies with quantitative study designs were included that were based on empirical data; (2) were conducted in all network settings, i.e., at international, state, regional, or community level; (3) had a research design utilizing both a socio-centric and an ego-centric network analysis; (4) analyzed only primary sources.

Studies were excluded if they: (1) examined health-promoting networks in which the promotion of PA was not mentioned or played only a minor role; (2) examined social networks between individuals; (3) examined networks dealing with a population suffering from a special disease. The search was limited to network analyses studies published in English. No limit was set regarding the year of publication or the publication status.

### 2.2. Search Strategy and Selection Process

Electronic databases and search engines (Web of Science, PubMed, and Scopus) were searched independently by three authors (IT, SR, CJ), with one author searching each database. Titles and abstracts of the search results were systematically screened to identify relevant articles. The term network analysis was combined with synonyms for collaboration and PA: (1) network analysis (“network analys*”); (2) collaboration (communit* OR collaboration* OR alliance* OR coalition* OR interorgani?ation* OR inter-organi?ation*); and (3) physical activity (“physical activit*” OR “health promotion” OR “health behave*” OR “public health”). The search strategy for all databases was conducted the same way, adapting the terms to the specific requirements (see Table S1). The final search was conducted in June, 2021. In addition, the references of the included studies were manually screened to identify any additional eligible studies that were not captured by the electronic search. Of the relevant studies, a list was compiled; each author (IT, SR, CJ) read through the full text and decided independently whether the paper met the inclusion criteria. There was uncertainty as to whether they should be included for eight articles, as the networks being investigated were only indirectly related to PA. Discrepancies and ambiguities were clarified in a discussion within the team (IT, SR, CJ, PB, HW). The reasons for excluding the studies were documented.

### 2.3. Data Extraction

A detailed DE template was developed to extract data from each study systematically. The DE was adapted to capture all relevant characteristics of studies that conducted network analyses, using the following categories: authors, network setting, aim, type of analysis, number of networks, type of nodes, type of ties, and network concepts/parameters used. Details from each study included in the systematic review were extracted independently and in duplicate by two authors. Subsequently, the two files were checked for differences and transformed into one file. Any differences were resolved through discussion, while a third author verified the information to counteract bias or error.

### 2.4. Quality Assessment

To assess the quality of the included studies, a QA tool was developed. Several items from existing QA tools [50,51,52,53] were used to develop a tool that fitted studies utilizing network analysis. The items included several network-specific aspects, such as a clear description of the analyzed network(s) and its (their) boundaries and a precise statement of how network data was collected. Since missing data can result in missing links between organizations in the resulting network [47], a high participation rate is critical and should comprise at least 75% of the organizations [54,55,56,57]. Since the respondent’s personal view of the organization’s relationships influences results [47], it is essential to declare who completed the survey.

The final QA tool included 11 unweighted items that enabled a rating of the studies. Every item was based on a question that can be answered with Yes, No, or Cannot determine (for details, see Table S2). Two authors independently evaluated the quality of the studies. In case of discrepancies, another author was consulted, and there was discussion until a consensus was found.

## 3. Results

### 3.1. Selection Process

**Figure 1:**
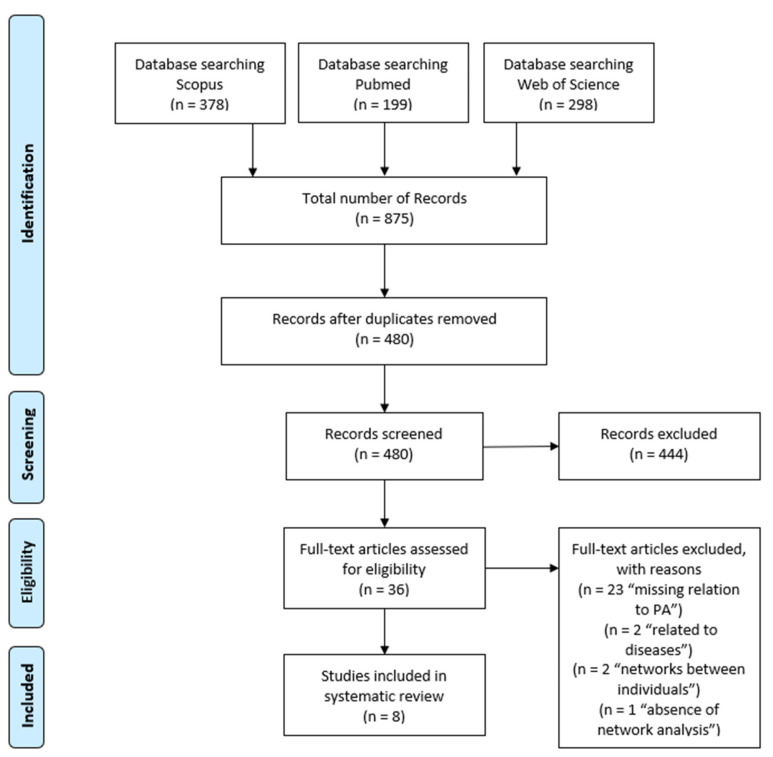
PRISMA flow chart of included studies.

In three databases, a total number of 480 articles was found. Finally, eight studies were considered eligible. The flow diagram outlines the selection process (see Figure 1).

### 3.2. Study and Network Characteristics

A summary of extracted data is given in Table 1. All of the networks were involved in the promotion of PA, most networks additionally focused on healthy lifestyles, active living, or included healthy eating. All studies were conducted in the Americas, the majority in Brazil (*n* = 3) and Canada (*n* = 3). Most of the studies considered networks at the level of a community, region, or a compound of counties (*n* = 4), but networks were also analyzed at the state (*n* = 2) and national level (*n* = 2).

The majority of studies aimed to describe network structure and examine relationships among involved organizations, thereby assessing the network. Moreover, the studies aimed to investigate differences between the networks. Other aims were to identify subgroups, roles, central and isolated actors, and to describe gaps and barriers in the network. Two studies either focused on the association between one kind of relationship and structural characteristics or one kind of relationship and organizational attributes [58,59]. Buchthal et al. aimed to supply a model for assessing other collaboratives, and Yessis et al. aimed to test the method of network analysis by evaluating a network [60,61].

All studies analyzed networks as a whole, and all studies conducted descriptive analysis. No study aimed for a longitudinal analysis or focused on aspects of network foundation. Three studies applied correlational analysis, and four studies utilized ERGMs for explanatory analysis. One study applied a discriminant function analysis to explain the impact of network centrality on PA promotion policy use [62].

Most studies analyzed a single network (*n =* 7). One study included two networks. The number of network organizations considered ranged from 22 to 52 organizations.

A plethora of organizations represented nodes for network analysis. While organizations from the health sector were dominant, organizations from education, sport and recreation, research, and various community organizations, were analyzed. Collaborative integration referred to the various degrees of collaboration; this was the most often analyzed relationship, considered in six studies. However, different terms such as intensity, collaboration, interaction, or involvement were used in several studies. In the following, it is referred to as collaborative integration. Data on various levels of collaborative integration were collected; the majority of studies cited the questionnaires by Slonim et al. or Provan et al. [37,63]. Buchthal et al., Loitz et al., and Meisel et al. used seven levels, including “not linked or integrated at all, communication, cooperation, coordination, collaboration, partnership and fully linked or integrated” [58,60,62] (p. 21). Yessis et al., Brownson et al., and Parra et al. set five levels of collaborative integration [59,61,64].

Further essential types of ties were importance and frequency of communication, mentioned in four studies. Importance describes the relevance of the other organizations or agencies in the network. Frequency of communication reports how often organizations recorded contact with each other, e.g., daily or monthly contact through meetings. Funding, i.e., the exchange of money, goods, or services between agencies, was reported by two studies and described whether and which organizations within the network received funding, sent funding, or both. Network-specific parameters and methods used in the studies are discussed together with key findings (see below).

### 3.3. Quality Assessment

Results of the QA for the included studies are summarized in Table 2. The participation rate was more than 75%, except for one study. The best quality rating for the included studies was ten out of eleven criteria, and the worst quality rating was seven criteria met, indicating a good quality for most studies. The most substantial deficits concerning the criteria were a lack of a description of how data were collected and a precise definition of the notions of health promotion and PA. Only two of the eight studies clearly defined PA, health promotion, active living, or similar terms. An appropriate definition of SNA was given by five of the eight studies. Equally, five studies mentioned ethical issues. Most criteria were fulfilled by Loitz et al., Buchthal et al., and Parra et al. [59,60,62]. The remaining studies fulfilled eight of the required eleven criteria.

### 3.4. Key Findings

In the following, the key findings of the studies reviewed are summarized. Following the categorization of Luke and Harris, findings on a network level are presented; afterwards, the findings with regard to the individual level (network actors) are described [26]. Finally, determinants of network outcome are discussed.

#### 3.4.1. Network Level

All included studies used the parameter of density to describe the networks. The studies reported low to moderate density scores with many ties not being realized (between 0.44 and 0.003) [45]. The results indicate that especially relations of a higher level of integration, such as collaboration and partnership, showed low density scores. Loitz et al. reported a moderate density of the coordination network, while the partnership network representing formal relations between agencies was only loosely connected [62]. Yessis et al. also found that the density of the network was lower when ties were closer (0.186 (awareness) to 0.027 (collaboration)) [61]. In the cooperation network, analyzed by Buchthal et al., almost all agencies were connected through multiple links, resulting in a density value of 0.42 [60]. Considering collaboration, a type of tie subordinated to cooperation, the network fragmented into three clusters and ten completely isolated actors (density value of 0.11). Two studies investigated funding. Density in terms of funding relationships among networks revealed low interconnectedness with values between 0.07 and 0.20 [60,62]. Seven studies considered (degree-/betweenness) centralization. Five networks showed a decentralized structure, i.e., a low to moderate range [59,60,61,62,65], whereas two networks showed a high degree of centralization, indicating a few powerful organizations influencing the network [59,66].

#### 3.4.2. Individual Level

Betweenness centrality, indicating information control and (potential) gatekeepers within the network, was the most analyzed parameter at the individual level used in six studies. This parameter was—if stated—generally low to moderate. Concerning degree centrality, Loitz et al. identified one central actor, that is, Alberta Tourism Parks and Recreation, a ministry at the state level in the Canadian PA promotion network, whereas Buchthal et al. identified two central actors at a state and county level in Hawaii (i.e., the state department of health and the nutrition and PA coalition agencies) [60,62].

In general, closeness centrality of members was higher the longer they participated in the network. Brownson et al. identified three community/governmental health organizations and one research organization as key players in Brazil [64]. Meisel et al. showed the central players in the Colombian network belonged to the sports and recreation, government, and security sectors [58]. The health sector in this bottom-up established network is not most central due to the multisectoral, self-organized network structure. Furthermore, structural equivalence is apparent when organizations of the same type (e.g., schools) are more likely to form a link than organizations from different sectors. Parra et al. examined specific sectors and found that research organizations were more likely to be affiliated with other research organizations of the same type, while practice organizations were less likely to collaborate with each other [59]. The network studied by Brownson et al. showed little structural equivalence—in contrast, the network studied by Meisel et al. showed a positive value of structural equivalence [58,64].

#### 3.4.3. Subgraph Level

At the subgraph level, organizations were found to have a positive tendency for transitivity; that is, organizations tended to form closed triads with other network members, and thus they cooperate in small groups rather than at the individual level [58,64,66]. In another network, educational organizations displayed closed triangles concerning fundraising [65].

#### 3.4.4. Determinants of Network Outcome

Network outcomes can be influenced by parameters like the maturity of a network or contextual conditions (e.g., perceived barriers). The duration of the investigated network was assessed in four studies. Buchthal et al. examined a three-year-old network, and the network analyzed by Yessis et al. existed for five years [60,61]. The network of Barnes et al. had persisted 12 years at the time of the investigation, and the most mature network was 38 years old [58,65]. Furthermore, Meisel et al. discovered that actors who had participated in the network for a longer period were less integrated or perceived as important [58]. According to the authors, established organizations seek to maintain the actual status rather than spend energy forming new ties or launching innovations.

The most common and frequently reported barrier was bureaucracy [58,59,64], followed by differing goals or agendas of organizations [58,60], lack of time [58,59,64], geographical distance [59,60], inability to identify appropriate collaborators, and costs of collaboration outweighing benefits [59,65]. In the study of Brownson et al., further barriers included interorganizational policies and inadequate previous experiences, whereas Parra et al. reported past experiences and interagency policies [59,64]. However, past experiences had a negative effect on forming partnerships in Brazil and a positive effect in Colombia [59]. In the study of Meisel et al., 40.9% reported no limiting factor, and 4.54% indicated a lack of formal agreements [58].

## 4. Discussion

In this review, interorganizational networks that explicitly promote PA were examined. The analysis revealed that there is limited knowledge about the structure and functioning of these networks so far. To date, eight studies have conducted SNA in PA promotion, and the paucity of data available makes it difficult to generalize or draw comparisons.

The first aim of this review was to explore structural characteristics and mechanisms of interorganizational networks promoting PA. With regard to structural cohesion, density was the most often analyzed parameter. A low to moderate density score was found across the reviewed studies. The included network analyses showing low densities indicated low levels of cooperation. However, the low to moderate density scores indicate a potential for closer ties to be established in collaboration and partnership networks. Higher network density could, for example, cause weaker informational links to turn into stronger, more formal ties and could also increase the speed of information transmission across a network [34].

Otherwise, a higher density score is not always advantageous since the time needed to intensify the relationships could be a burden for participating members, or the focus of individual organizations differs to the extent that a close linkage is not necessary [47]. It also became apparent that high density in organizational networks can represent a weakness—this can reduce efficiencies and lead to an overload of central nodes in highly centralized networks [17,37,67]. Buchthal et al., Loitz et al., and Yessis et al. revealed that organizations tended to work side by side rather than as partners and that close partnerships were not very common [60,61,62]. This could also be observed in health-promoting networks with no specific focus on PA [68,69]. In addition, in a local network, it could be shown that despite the low density in the community network, it was not intended to promote densification of relationships since any actor could reach almost another within the network [66].

Density is typically lower for more extensive networks due to the complexity of relations [29]. Contrary to this assumption, the results of Parra et al. indicated that the Colombian network, including more actors (*n =* 45), showed a higher density and was thus more cohesive than the smaller network of Brazil (*n =* 28) [59]. In this context, the Colombian network was characterized by a higher degree of centrality, i.e., a few organizations represent the key players, than the Brazilian network [59].

Another possible explanation for the low density scores could be cliques. Cliques depict a group of at least three interconnected actors [26]. Working in cliques or clusters with specific roles and activities will naturally lead to a lower density but does not necessarily indicate lower effectiveness, e.g., after removing a critical set of nodes, the structure of the Ciclovía network was still robust [58,70]. Cliques of three or more organizations develop strong ties among themselves and can increase effectiveness compared to a network in which everyone works closely with everyone else [47]. However, a prerequisite for this effectiveness is that it is transparent to all actors whose members belong to which clique and, most importantly, whether the network’s goals can be further pursued through the clique structure [47].

Another aspect of our review dealt with network centralization. The investigated networks of Sao Paulo [66] and Colombia [59] showed a relatively high centralization score. A possible explanation could be that the network of Sao Paulo was implemented by the local government, with one actor from the public sector claiming 40% of the collaboration and partnership ties [66]. Likewise, the network of Colombia showed that 77% of the organizations were from the government sector [59].

In a young network, and especially at the beginning of the network formation process, it seems plausible that the network needs to be centralized. In contrast, more mature tobacco control networks were found to be more centralized [35,71]. There has been little experience of cooperation between actors from different sectors in relation to PA promotion at the municipal level; hence the young network had a low centralization [60]. Accordingly, the networks in the present cases existing for three to twelve years showed an increased centralization.

Regarding types of collaboration, two studies examining collaborative integration revealed that the higher the integration, the lower the centralization [60,61]. Contrarily, a study showed that the more intense a partnership, the higher the centralization of a network [62]. However, the factors defining the centralization of networks remain unclear, and the findings cannot be generalized since initiating organizations, boundary setting, or governmental defaults, among others, are important determinants that influence network centralization. Long-term studies are critically required to map the development of networks over time.

This review also revealed that homophily (i.e., the tendency of organizations to collaborate with other organizations in the same field) seems to be a widespread mechanism among PA promoting networks [59,60,64,72]. In the field of PA promotion, it seems natural that organizations of the same type are more strongly connected because of the similar nature of work and the comparable organizational settings [29,73]. Cooperation with actors belonging to the same type could also be observed in networks of healthy lifestyle promotion [17,69,74]; this circumstance could be avoided, for example, by stringent leadership and coalition-promoting behavior, e.g., by state governments [69]. There was also a tendency for transitivity in three networks—meaning that organizations tended to cooperate and build partnerships in small groups rather than on an individual level [58,64,66]. In these closed triangles, they shared information, values, and norms and assisted in problem-solving. Within one network, organizations formed complete cliques based on fundraising [65]. However, this reinforces the possibility that some actors are entirely isolated from some form of integration networks [19]. For example, it was found that actors are more likely to foster connections that meet their primary organizational concerns rather than overarching health issues [15].

Essential factors for effective collaboration at the community level include the diversity of a network; in tobacco control networks, it has already been shown that a transdisciplinary network is promising [75,76]. The contact to more distant ties offers new input and the contribution of other ideas [77,78]. Therefore, it can be beneficial for the development of networks to integrate stakeholders from other disciplines, e.g., from industry, advertising, sport science, to draw attention to health promotion programs and reach a larger target audience [79]. Transdisciplinary cooperation is also supportive in terms of translating scientific findings and implementing and embedding them into community health programs [80,81].

The second aim of this review was to identify the role and characteristics of the organizations involved, as well as potential barriers to cooperation among individual actors in PA promoting networks. Most of the included studies identified governmental health organizations as key players in their networks. However, actors from other sectors or agencies took bridging roles in the networks, such as a youth organization like the YMCA [60] or other non-profit organizations [61,65]. State-level, as opposed to community-level organizations, acted as gatekeepers, and community-level organizations served as gatekeepers for smaller organizations. Moreover, organizations from the educational sector occasionally showed themselves to be in influential positions [62,65].

These results showed that governmental, health, and educational agencies often play central roles in networks promoting PA. Organizations of the same type seemed rather more likely to work together in terms of cooperation, especially research organizations.

Multiple challenges of collaborative integration among different organizations arose within a network; bureaucracy, organizational structures, and interorganizational policies presented barriers in collaboration [58,59,64]. The dynamic structure of linkages rendered it difficult in centralized networks to obtain information about what other institutions were working on in the field of PA promotion [59,62]. Potential barriers also included the differential or incompatibility of network goals, as the vision of a network did not necessarily reflect an organization’s vision [62]. Furthermore, intensive collaboration required an enormous investment of time [58,62,64,82].

Overall, the quality of the studies using SNA to analyze PA promoting networks is good. While most studies are descriptive, some applied explanatory designs and methods. However, the number of studies is low, and the networks examined in the included studies had heterogeneous settings and various influencing variables. This hampers a direct comparison between the investigated networks and the drawing of general conclusions.

### Limitations

There are potential limitations to the results of this review. Firstly, regarding the self-developed quality assessment, it may be possible that not every aspect that predicts the quality of network analysis studies is included in the QA template. Furthermore, it is uncertain that the chosen boundary of 75% for the participation rate [54,55,56,57] was the best-accepted value. For example, frequent follow-up calls and face-to-face interviews could result in response rates of close to 90% [47]. However, the QA provides an opportunity to use it as an orientation for future assessment of network studies. Secondly, some relevant information from the network studies may not have been considered in the DE. Thirdly, no studies with network analysis were included, in which PA promotion was an additional component within a program, which may have led to the exclusion of relevant studies. Lastly, the search was limited to studies published in English, which might have also led to the omission of relevant literature.

Moreover, the results of the network analyses of the included studies may be interpreted with some limitations. Although all studies represented a cross-section of the current network, the limitation of this method is acknowledged by various authors [58,62,64,68]. Furthermore, while all studies reported density, a comparison is challenging since density depends on network size. For future studies, reporting the average degree would make it easier to compare different networks [83].

Additionally, networks constantly change within and between organizations [62], resulting in studies representing snapshots that might not necessarily reflect the actual network structure. It could be observed whether, on the one hand, the health behavior of the investigated population has changed or, on the other hand, the network itself has changed—for example, through intensified partnerships. However, Brownson et al., McCollough et al., and Meisel et al. announced a follow-up analysis and could be the first to submit a longitudinal study for networks promoting PA [58,64,68]. Interestingly, in five studies, the key players corresponded with the institutions that funded the respective study [58,59,60,62,64]. The fact that key players funded the study concerned in five cases might limit the objective work and hinder the potential influence of other organizations. For further SNA research, own models should be designed for networks that deal with PA implementation. Thus, hypotheses can be verified with an appropriate theory [84]; with appropriate models, the results of the network analysis can be evaluated, and studies could be compared more easily.

## 5. Practical Implications

Besides an overview of network-analytic studies on interorganizational networks in PA promotion, the study contributes to this field of research by providing a specific QA template. For future research, the DE and QA templates offer the opportunity to assess current research and provide guidelines for future research. Due to the increasing sedentary behavior and the lack of PA in the population, it is of utmost importance for PA promotion networks to work effectively; therefore, this review highlighted some aspects that facilitate interorganizational collaboration by, for example, identifying common sources of problems and offering approaches to tackle them.

To establish effective network ties, it is crucial to identify key players and involve them actively. Essentially, if the network is characterized by few key players having great influence, the structure can be vulnerable [85]. Being aware of gatekeepers could assure the diffusion of the idea of PA promotion, information, and likewise innovations. This could indicate the future development of networks [47]. Initial leadership at the beginning of an emerging network confers the benefits of an intact infrastructure (e.g., providing online tools) that supports organizations to build mutual relationships and initiate joint projects [61]. There may be potential for using online social networks to foster community dynamics of PA programs [86].

Despite a tendency to homophily [59,60,61,69], transdisciplinary cooperation is in many cases enriching, enabling synergies, and providing valuable opportunities to collaborate to promote PA and health [34,87]. It is not only organizational differences that pose a barrier to collaboration but also physical distance, as Buchthal et al. showed for state and county agencies and voluntary agencies located in different counties [60]. This adds to prior research showing that collaborative ties are stronger when partners were physically close [88]. Regular meetings, conference calls, or video conferencing are valuable tools for network development to overcome deficits resulting from a physical distance and be relied on to maintain effective networks. According to Barnes et al., a greater number of ties for information exchange was caused by bi-monthly working group meetings and regular electronic correspondence [65].

Incompatibility of goals can be a limiting factor. Organizational visions, missions, and roles can be different from the visions or goals of the network. Such incompatibility may prevent organizations from sharing resources and collaborate as partners [62]. Using “formal agreements”, goals can be determined in advance, giving every organization the possibility to work in its sector, find its part in the network, and provide consent in overall health aspirations [58,89]. Hence, the apprehension of interorganizational relationships leading to a loss in autonomy and depletion of own resources can be counteracted [26]. Future interorganizational collaborations should therefore work on a distinct objective already during the development of the network.

Moreover, network outcomes should be examined with reference to the maturity of the network. McCullough et al. figured out that more extended participation in the network is associated with greater closeness centrality and overall value [68]. The overall value of an organization was also mentioned as a key aspect predicting collaboration by Brownson et al. and Parra et al. [59,64]. The opposite findings of Meisel et al. refer to the six states of network maturity of Batonda and Perry: searching, starting, development, maintenance, termination, and dormant (and re-activation) processes [58,90]. Accordingly, organizations in the maintenance status could act less enthusiastically and innovatively. Nevertheless, established organizations are essential as a key resource for new policymakers and practitioners by learning from the experiences of these institutions [64].

In conclusion, as Provan et al. mentioned, it stands to reason to analyze special examination questions relating to the maturity of the network. Results of an SNA should be associated with the maturity or the state of network development to explain network outcomes more precisely [37]. In this domain, further research would be useful.

## 6. Conclusions

This review exemplified the network structures investigated in eight studies that focused primarily on the promotion of PA. The identified networks showed a low density primarily. Due to their different survey methods, the networks were difficult to compare; nevertheless, especially at the beginning of a network formation, increased integrating activities are needed to strengthen the network ties. A low to high centrality in the integrated networks was found. The level of collaborative integration seemed to be mainly a time factor, i.e., in young networks the cooperation was side by side rather than in teams (collaboration).

Collaboration in interorganizational networks requires more than information sharing; in particular, founded networks that aim to promote PA encounter many challenges; their target group range across all populations—on the one hand, they should work preventively, on the other hand, they should also reduce ongoing inactivity and resulting NCDs. In relation to the established research area on tobacco control networks, it appears that there are hardly any comparative components and experiences available so far, which existing PA promotion networks can orientate on. Therefore, it is crucial to evaluate PA promotion networks over time to understand how, for example, different levels influence the network’s processes. More long-term studies are required; hence, longitudinal data can provide the opportunity to examine network evolution [47,77]. Furthermore, it should be empirically recorded through qualitative methods which activities improve the outcome, i.e., the effectiveness and efficiency of the network.

SNA methodology in health-promoting networks provided unique insights into how community partnerships work together to promote PA that could not be readily exposed through conventional surveys and statistical analysis [69]. SNA is a promising approach that provides benefits not only for research but also for the practice of PA promotion and structural interventions. Transdisciplinary cooperation across different sectors engenders opportunities for successful and sustainable health interventions and promotes active lifestyles, leading to the physiological and mental well-being of the population.

## Figures and Tables

**Table 1:** Data extraction for included studies.

Authors	Network Setting	Aim	Type of Analysis	No. of Networks (Organizations)	Types of Nodes	Types of Ties	Network Concepts/Parameters
Andrade et al. (2018)	District of Sao Paulo, Brazil (community level)	Assessment of network structure, describe factors associated to establish collaboration or partnership ties	Descriptive, explanatory (ERGM)	One network (*n =* 32)	Actors from open streets, community clubs, social organizations, and public sector	Collaborative integration, contact, distance	Density, betweenness-/in-degree-/out-degree- centrality, transitivity, centralization,
Barnes et al. (2010)	One region in Canada (community level)	Assessment of network structure, identification of types of ties	Descriptive	One network (*n =* 31)	Community-based, non-profit and public actors (education, government, recreation, health, social services)	Collaborative integration (resources, information, fundraising, marketing)	Density, centralization, cliques
Brownson et al. (2010)	Brazil, USA (national level)	Assessment of network structure, roles, gaps and barriers	Explanatory (ERGM)	One network (*n =* 28)	Actors from research, education, promotion of PA in practice settings, actors developing and implementing policy	Collaborative integration, leadership, contact, importance	Density, closeness-/betweenness-/ in-degree-/out-degree- centrality, transitivity, centralization, structural equivalence
Buchthal et al. (2013)	Hawaii, USA (state level)	Assessment of network structure and identification of key roles; provision of a model for evaluation	Descriptive	One network (*n =* 23)	Department of health, nutrition and physical activity coalition agencies, other government agencies, voluntary organizations, health insurance companies	Collaborative integration, communication, funding, importance	Density, betweenness centralization/centrality
Loitz et al. (2017)	Province of Alberta, Canada (state level)	Assessment of network structure, examination of PA-policy use	Explanatory (discriminant function analysis)	One network (*n =* 27)	Actors from education, health, recreation, community, human services, transportation, fitness, child services or programming	Collaborative integration, funding	Density, degree-/betweenness centralization, degree-/betweenness centrality
Meisel et al. (2014)	Bogotá, Colombia (community level)	Identification of agencies, roles, structure, subgroups; relationship between structural characteristics and integration	Explanatory (ERGM)	One network (*n =* 22)	Actors from transport and urban planning, marketing services, research and academy, sports and recreation, government, health, security, education, environment	Collaborative integration, relationship, contact, importance, leadership	Density, closeness-/betweenness-/in-degree-/out-degree-centrality, reciprocity, structural equivalence
Parra et al. (2011)	Colombia and Brazil (national level)	Description and comparison of predictors of collaboration	Explanatory (ERGM)	Two networks: Brazil (*n =* 28), Colombia (*n =* 45)	Actors from the government sector and non-government sector (research, education, policy, practice)	Collaborative integration, importance, distance	Density, centralization
Yessis et al. (2013)	School setting in Canada, Ontario (community level)	Testing the method of network analysis for evaluating the program *Spark*	Descriptive	One network (*n =* 52)	National, provincial, regional, local organizations from urban and rural settings (health, education, recreation, public service, community/citizen groups)	Collaborative integration	Density, centralization, centrality, degree-/betweenness centrality

**Table 2:** Quality assessment for included studies. Yes = +; No = −; Cannot determine = 0.

Authors	1. Aims of the Research	2. Boundary Setting/Actor Identification	3. Participation Rate ≥ 75%	4. Data Collection	5. Description of Investigated Network	6.1. Definition of Health Promotion and Physical Activity	6.2. Definition of Social Network Analysis	6.3. Definition of Variables	7. Same Mode of Data Collection for all Subjects	8. Ethics	9. Findings	Total Number Yes/No/Cannot Determine
Andrade et al. (2018)	+	+	0	+	+	−	−	+	+	+	+	8/2/1
Barnes et al. (2010)	+	+	+	+	+	−	+	+	−	−	+	8/3/0
Brownson et al. (2010)	+	+	+	+	+	−	−	+	−	+	+	8/3/0
Buchthal et al. (2013)	+	+	+	+	+	+	+	+	+	−	+	10/1/0
Loitz et al. (2017)	+	+	+	+	+	+	+	+	+	+	+	11/0/0
Meisel et al. (2014)	+	+	+	−	+	0	0	+	+	+	+	8/1/2
Parra et al. (2011)	+	+	+	0	+	0	+	+	+	+	+	9/0/2
Yessis et al. (2013)	+	+	+	+	+	−	+	+	−	−	+	8/3/0
Total number Yes/No/Cannot determine	8/0/0	8/0/0	7/0/1	6/1/1	8/0/0	2/5/3	5/2/1	8/0/0	5/3/0	5/3/0	8/0/0	

## Data Availability

Data sharing is not applicable to this article.

## Supplementary Materials

The following are available online at https://www.mdpi.com/article/10.3390/ijerph18147306/s1, Table S1: extended search term, Table S2: detailed quality assessment.
