# Supplementary material for: Interorganizational Networks in Physical Activity Promotion: A Systematic Review
Source: Int J Environ Res Public Health. 2021 Jul 8;18(14):7306. doi: 10.3390/ijerph18147306 (PMC8306254; doi:10.3390/ijerph18147306)
Supplement: Supplementary file 1 [file ijerph-18-07306-s001.zip › Table_S1_Searchterm_submission.pdf]

Supplemental Material

Table S1. Extended search term.

**Database**	**Search term**
Web of Science	(TS=("network analys*") AND TS=(communit* OR collaboration* OR alliance* OR coalition* OR interorgani?ation* OR inter-organi?ation*) AND TS=("physical activit*" OR "health promotion" OR "health behavi*" OR "public health")) *AND* **LANGUAGE:** (English)
Pubmed	((("network analysis"[Title/Abstract] OR "network analyses"[Title/Abstract])) AND (communit*[Title/Abstract] OR collaboration*[Title/Abstract] OR alliance*[Title/Abstract] OR coalition*[Title/Abstract] OR interorganisation*[Title/Abstract] OR interorganization*[Title/Abstract] OR inter-organisation*[Title/Abstract] OR inter-organization*[Title/Abstract])) AND ("physical activity"[Title/Abstract] OR "physical activities"[Title/Abstract] OR "health promotion"[Title/Abstract] OR "health behavior"[Title/Abstract] OR "health behaviour"[Title/Abstract] OR "public health"[Title/Abstract])
Scopus	(**TITLE-ABS-KEY**("network analysis") AND **TITLE-ABS-KEY**(communit* OR collaboration* OR alliance* OR coalition* OR interorganisation* OR interorganization* OR inter-organisation* OR inter-organization*)AND **TITLE-ABS-KEY**("physical activit*" OR "health promotion" OR "health behavi*" OR "public health"))
