# Supplementary material for: Interorganizational Networks in Physical Activity Promotion: A Systematic Review
Source: Int J Environ Res Public Health. 2021 Jul 8;18(14):7306. doi: 10.3390/ijerph18147306 (PMC8306254; doi:10.3390/ijerph18147306)
Supplement: Supplementary file 1 [file ijerph-18-07306-s001.zip › Table_S2_QA Tool for SNA Studies_submission.pdf]

Supplemental Material

Table S2. Quality assessment tool for social network analysis studies.

Name of author(s):
Year of publication:
Name of paper/study:

Appraisal question	Criteria for answers			Additional notes
	Yes	No	Other (CD, NR, NA)*	
1. Was there a clear statement of the aims of the research?				- **What was the goal of the research?**<br>- **Why was it thought important?**<br>- **Its relevance**
2. Was there a clear description of the Boundary setting / the Actor identification?				- **Of networks (nominalist/realist strategy)/ Organizations (how were the participating organizations selected?) (snowball method?)**
3. Was the participation rate of eligible actors at least 75%?				- **> 75 %**
4. Was there a clear statement how data were collected (directly from the subjects as opposed to a proxy)?				- **Subjects (management role, CEO, external (expert survey))**<br>- **Proxy (documents, database)**
5. Was the investigated network clearly described?				- **Was it formed unintended or strategically?**
6. Definitions<br>6.1<br>Was an acceptable definition of health promotion/activity used in the study?				- **Wide definition of sport (additional interventions possible, e.g., nutritional advice, psychological interventions, counseling)**
6.2<br>Was an acceptable definition of social network analysis or a similar theoretical foundation used in the study?				- **Definition of social network analysis**
6.3<br>Was an acceptable definition of variables used in the study?				- **Were the investigated variables clearly defined? (e.g., cooperation, partnership)**<br>- **Were the network measures clearly defined? (e.g., degrees of network centrality. Valid, reliable?)**
7. Was the same mode of data collection used for all subjects?				- **Face-to-face interviews, telephone interviews, questionnaires, tape recordings, video material, notes, etc.**

8. Have ethical issues been taken into consideration?				- **Discussion of how the anonymity of participants/sources was protected**<br>- **Discussion of confidentiality of data and procedures for protecting**
9. Is there a clear statement of findings?				- **Are the findings explicit?**<br>- **Is there an adequate discussion of the evidence both for and against the researchers' arguments?**<br>- **Has the researcher discussed the credibility of their findings? (e.g., triangulation, respondent validation, more than one analyst)**<br>- **Are the findings discussed in relation to the original research question?**
Quality Rating:				
Rater #1 initials:				
Rater #2 initials:				

* CD, cannot determine; NA, not applicable; NR, not reported
